# Safety and Effectiveness of Sodium–Glucose Cotransporter 2 Inhibitor Combined with Medical Nutrition Therapy for Hyperglycemia in Acute Stroke: A Retrospective Study

**DOI:** 10.3390/metabo12010025

**Published:** 2021-12-28

**Authors:** Takahisa Mori, Kazuhiro Yoshioka, Yuhei Tanno, Shigen Kasakura

**Affiliations:** Department of Stroke Treatment, Shonan Kamakura General Hospital, Kamakura City 247-8533, Kanagawa, Japan; y.kazuhiro12@icloud.com (K.Y.); yxip01@icloud.com (Y.T.); kasakura@med.kitasato-u.ac.jp (S.K.)

**Keywords:** acute stroke, carbohydrate, diabetes, energy restriction, hyperglycemia, nutrition therapy, SGLT2 inhibitors

## Abstract

Hyperglycemia, a predictor of poor clinical outcomes in acute stroke, must be lowered safely and promptly. We investigated the safety and effectiveness of sodium-glucose cotransporter 2 inhibitors (SGLT2is) combined with medical nutrition therapy in lowering blood glucose levels. This retrospective study included stroke patients admitted between 2014 and 2019, who (1) had glycated hemoglobin ≥6.5%, blood glucose level ≥ 11.1 mmol/L at admission, (2) took their diet and drugs orally during hospitalization, (3) underwent SGLT2is pharmacotherapy after admission, and (4) underwent a fasting blood glucose (FBG) test on day 7. Patients were provided with a moderate-carbohydrate diet combined with total energy restriction. We assessed the achievement of FBG < 7 mmol/L on day 7 and the need for sulfonylurea or a long-acting insulin analog (LIA) treatment during hospitalization, which carries a risk of hypoglycemia. Fifty-one patients met our inclusion criteria. Of them, 33 (64.7%) achieved the target FBG on day 7. Only eight patients were treated with a small dose of LIA; however, no patients required sulfonylurea. No dehydration occurred. SGLT2is combined with a moderate carbohydrate- and energy-restricted diet achieved the target FBG level safely, effectively, and promptly in mild stroke patients with oral ingestion.

## 1. Introduction

The treatment regimen for hyperglycemia in acute stroke must be urgently improved because it is related to poor functional outcomes [1,2]. However, safe and effective treatment for hyperglycemia has not been established. Severe hypoglycemia following attempts such as intensive insulin treatment has been reported [3]. Medical nutrition therapy is the first-line treatment for type 2 diabetes mellitus (T2DM) during hospitalization. In Japan, T2DM patients receive a total energy-restricted (TER) diet during hospitalization [4]. In addition, reducing overall carbohydrate (carb) intake in T2DM patients according to recommendations [5] has been the best support for improving hyperglycemia [6,7]. Oral medications for T2DM are essential for stroke prevention. Patients with T2DM at high risk for cardiovascular events who received empagliflozin, an inhibitor of sodium-glucose cotransporter 2 (SGLT2), had a lower death rate from all causes [8]. However, few studies have reported on the safety and effectiveness of SGLT2 inhibitors (SGLT2is) in acute stroke. This may be because SGLT2is have a diuretic action that can lead to dehydration, an adverse effect [9,10]. SGLT2i combined with a moderate-carb diet may promptly lower blood glucose (BG) levels in acute stroke [11]. Sulfonylurea (SU) or a high dose of insulin is commonly used for hyperglycemia at admission. Therefore, if SGLT2i combined with medical nutrition therapy can promptly lower BG levels without SU or a high dose of insulin, the treatment would be safe and effective for acute stroke patients with hyperglycemia. The purpose of this retrospective case series study was to investigate the safety and effectiveness of SGLT2is combined with a moderate-carb TER diet for improving hyperglycemia in acute stroke. 

## 2. Results

### 2.1. Primary Analysis

**Figure 1:**
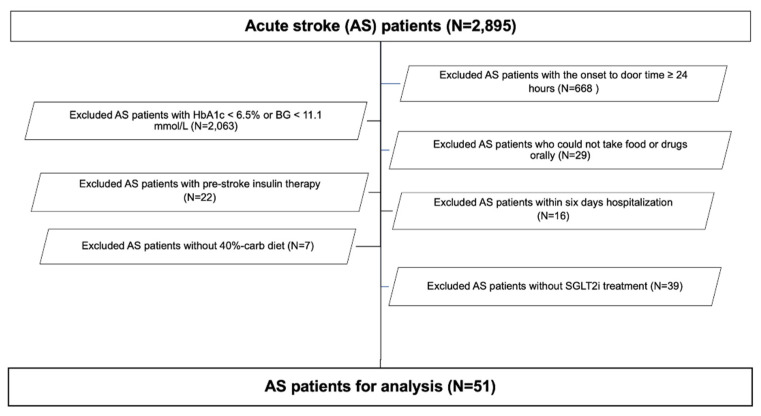
Flow chart of patient selection for the analysis. BG, blood glucose; carb, carbohydrate; HbA1c, glycated hemoglobin; SGLT2i, sodium-glucose cotransporter 2 inhibitor.

Of 2895 stroke patients, 51 met our inclusion criteria (Figure 1). Patient characteristics are summarized in Table 1, and stroke subtypes are shown in Supplementary Material Table S1. Among the three patients with cardioembolism, one underwent mechanical thrombectomy, and among 19 patients with small-vessel occlusion, one underwent intravenous tissue-plasminogen activator therapy. None of the three patients with hypertensive ICH underwent surgical hematoma evacuation. T2DM medications used prior to admission are summarized in Supplementary Material Table S2. The median TER was 1400 kcal/day (Supplementary Material Table S3), and the median daily carb intake was 140 g during hospitalization (Table 2). Three types of SGLT2i were used (Supplementary Material Table S4), and 42 patients (82.4%) took T2DM drugs (Supplementary Material Table S5). Eight (15.7%) of 51 patients were treated with a small dose of long-acting insulin analog (LIA), insulin degludec. Their median dose of LIA was six units only on day 7. However, no patients were treated with SU (Supplementary Material Table S5).

**Figure 2:**
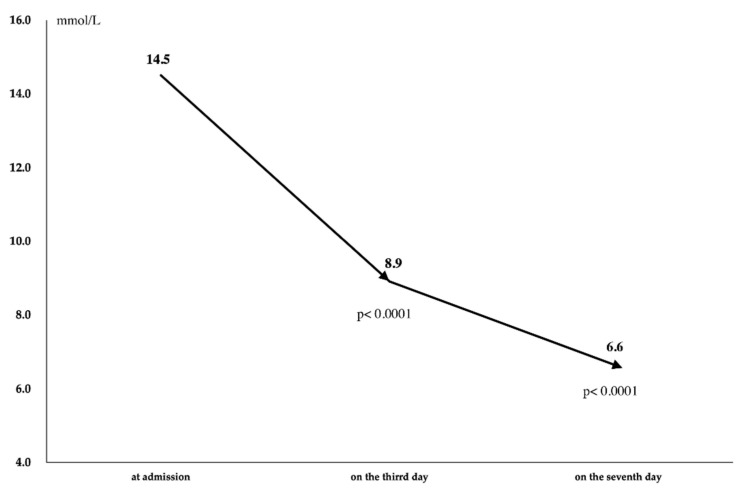
Serial changes of median blood glucose level.

BG levels decreased from admission to the third day and from the third day to the seventh day (Table 3, Figure 2), and 33 patients (64.7%) achieved the target fasting blood glucose (FBG) of <7.0 mmol/L on day 7 (Day-7 FBG < 7.0) while 18 patients did not achieve the target FBG on day 7 (Day-7 FBG ≥ 7.0) (Table 2). Median hematocrit (Hct) level decreased from 43.1% at admission to 40.6% on day 7 (Table 4). Dehydration occurred in no patients. 

Twenty-one patients (41.2%) showed a urine glucose score ≥4 at admission. In contrast, 46 patients (90.2%) showed a urine glucose score ≥4 on day 7 (Table 4). At admission, six patients (11.8%) showed a urine ketone score ≥1; no patients had a urine ketone score of ≥3. The median urine ketone score was zero at admission. In contrast, on day 7, 32 patients (62.7%) showed urine ketone score ≥1, 12 patients (23.6%) urine ketone ≥3. The median urine ketone score increased from 0 at admission to 2 on day 7 (Table 4). No patients presented with signs or symptoms of diabetic ketoacidosis (DKA). 

### 2.2. Secondary Analysis

There were no differences in BG levels at admission or in FBG levels on day 3 between Day-7 FBG < 7.0 and Day-7 FBG ≥ 7.0 groups; however, there was a difference in FBG levels on day 7 between the two groups (Supplementary Material Table S6, Supplementary Material Figure S1). BG level in the Day-7 FBG < 7.0 group significantly decreased from admission to the third day and from the third day to the seventh day. On the other hand, BG level in the Day-7 FBG ≥ 7.0 group significantly decreased from admission to the third day; however, the change was not significant from the third day to the seventh day (Supplementary Material Table S7). 

There were differences in age and the frequency of SU use before admission between Day-7 FBG < 7.0 and Day-7 FBG ≥ 7.0 groups; DPP4 inhibitors were more frequently used during hospitalization in the Day-7 FBG ≥ 7.0 than in the Day-7 FBG < 7.0 group (Supplementary Material Tables S8–S11). 

We adopted age at admission and SU use before admission as variable candidates for multiple logistic regression and confirmed their variance inflation factor (VIF) of less than 3 (Supplementary Material Table S12). Multiple logistic regression analysis showed that no SU use before admission was an independent variable of Day-7 FBG < 7.0 (Supplementary Material Table S13).

## 3. Discussion

SGLT2is combined with the 40%-carb-TER diet was safe and effective for improving hyperglycemia in acute stroke patients with oral ingestion. With this treatment, no SU was used, although a low dose of LIA was used occasionally. Many patients achieved the target FBG on day 7 without SU or a high dose of LIA carrying the risk of severe hypoglycemia.

Hyperglycemia induces endothelial cell dysfunction characterized by reduced antithrombotic properties and activation, defined by a decrease in the bioavailability of nitric oxide [12]. Inflammation is a crucial element in the pathogenesis of endothelial dysfunction as well as atherosclerosis, and the intake of foods with a low glycemic index might lead to a reduction in insulin resistance and consequent hyperinsulinemia [13]. Endothelial dysfunction may lead to vasoconstriction, thrombosis, and atherosclerosis [14].

Pioglitazone lowers the risk of recurrent major adverse cardiovascular events. However, this drug has been associated with an increased risk of heart failure [15,16] and bladder cancer [17] and is therefore not widely used. None of our patients had used pioglitazone before admission, and it was not used during hospitalization either. The 40%-carb-TER diet may contribute to not needing the addition of SU or reducing LIA dose and thus provides relatively safe treatment of hyperglycemia because SU or high-dose LIA may cause hypoglycemia. 

When BG level was ≥ 11.1 mmol/L and glycated hemoglobin (HbA1c) ≥ 6.5% at admission despite SU treatment prior to stroke onset, fewer patients achieved the target FBG. On the other hand, their FBG levels decreased from admission to the third day, and medical nutrition therapy was effective (online Figure 1). The effect of oral DM medications may be weak in patients presenting with hyperglycemia despite SU treatment. Chronic exposure to SU or glinide reduces insulin content and accelerates apoptotic *β*-cell death [18].

We administered SGLT2i in patients with estimated glomerular filtration rate (eGFR) ≥ and <60 mL·min^−1^·1.73 m^−2^ body surface area (BSA), and there was no difference in the FBG level on day 7 (Supplementary Material Table S7), although SGLT2is are less effective in lowering BG in patients with impaired renal function [19]. The preventive effect of SGLT2i on stroke may vary according to the level of kidney function [20]. The risk of fatal or nonfatal stroke was low in patients with eGFR 30 ≤ 45 mL·min^−1^·1.73 m^−2^ BSA [21]. The median eGFR in our patients was 68.2 mL·min^−1^·1.73 m^−2^ BSA, and the secondary preventive effect of SGLT2i in our patients might not be superior to that of patients with eGFR 30 ≤ 45 mL·min^−1^·1.73 m^−2^ BSA, although the BG level decreased promptly. 

Furthermore, euglycemic DKA in patients treated with SGLT2is has been reported [22]. In our study, 12 (23.6%) of 51 patients had urine ketone test scores ≥3 on day 7. They were asymptomatic; however, euglycemic DKA must be considered in the case of SGLT2is pharmacotherapy.

### 3.1. Limitations

Our study had several limitations. First, a small number of patients were included. Second, the study design was retrospective without a control group of patients with T2DM drugs except for SGLT2is combined with a 40%-carb-TER diet. Furthermore, we did not compare our treatment group with a low- or high-carb diet group, with or without SGLT2is. Third, because most patients were Japanese, the generalizability of the study outcomes to non-Japanese populations is uncertain; racial differences may exist in the efficacy of SGLT2is combined with a 40%-carb-TER diet for lowering FBG levels. Therefore, the reproducibility of our study is unclear, and a prospective study is warranted to establish that SGLT2is combined with a 40%-carb-TER diet can achieve the target FBG level more safely and promptly than conventional pharmacotherapy with a high-carb diet.

### 3.2. Conclusions

SGLT2is combined with the 40%-carb-TER diet achieved the target FBG level safely, effectively, and promptly. In mild stroke patients with oral ingestion, SU, or a high dose of LIA carrying the risk of severe hypoglycemia, may no longer be necessary. 

## 4. Materials and Methods

We included patients who (1) were admitted within 24 h of stroke onset between April 2014 and March 2019, (2) had a BG level ≥ 11.1 mmol/L (200 mg/dL), HbA1c level ≥6.5% at admission, and (3) underwent a FBG test on day 7. We excluded patients who (1) had undergone insulin therapy before stroke onset or (2) did not take their diet and drugs orally due to stroke severity during hospitalization. We diagnosed T2DM by a BG level ≥11.1 mmol/L combined with HbA1c ≥ 6.5% at admission. 

Target total energy (TTE) is based on the patient’s target body weight (TBW), which is calculated as follows [4]:

TBW = body height × body height × 22 kg/m^2^.
The TTE for the TER is calculated as follows [4]:
TTE = TBW × 25 kcal/kg.

A TER diet for hospitalization was determined (Supplementary Material Tables S1–S13). According to recommendations, we provided patients with a 40% (moderate) carb diet [5]. 

If a portable glucose meter indicated an FBG level ≥ 7.0 mmol/L (126 mg/dL) the day after admission, oral medications for T2DM were started. When the FBG level had decreased to <11.1 mmol/L (200 mg/dL) on the second or third day, SGLT2i therapy was initiated because hyperglycemia may cause osmotic diuresis. A LIA was administered when the FBG level was >13.8 mmol/L (250 mg/dL) on the day after admission. The initial LIA dose of 2 units was injected subcutaneously, and the LIA was discontinued when the FBG level had decreased to <5.6 mmol/L (100 mg/dL). Increases in LIA dosage depended on the attending physicians.

We carried out the Day-7 FBG test on the sixth, seventh, or eighth day after admission. Our target FBG level on day 7 was <7.0 mmol/L (126 mg/dL). We performed a urine strip test to check for glucose or ketones. Test scores ranged from 0 to 4. A value of zero indicates no glucose or ketones in the urine. 

### 4.1. Evaluation

We recorded the age and sex and evaluated anthropometric variables such as body weight, body height, BMI, TTE, daily carbohydrate intake, serum lipid level, serum creatinine level, eGFR, BG level at admission, FBG level on day 3 and day 7 after admission, achievement of the target FBG on day 7, HbA1c at admission, Hct level at admission and on day 7, urine glucose and urine ketones on admission and day 7, use of diabetic therapeutic drugs such as SU during hospitalization, and use of basal insulin therapy on day 7. If the urine ketone score was high, the DKA signs, polyuria, polydipsia, dyspnea, nausea and vomiting, abdominal pain, or weakness [23,24] were assessed. In addition, Hct levels were measured to determine dehydration during hospitalization. For the secondary analysis, variables were compared between the Day-7 FBG < 7.0 and Day-7 FBG ≥ 7.0 groups, and independent variables at admission associated with Day-7 FBG < 7.0 mmol/L were identified. 

### 4.2. Statistical Analysis

Non-normally distributed continuous variables were expressed as medians and interquartile ranges. The chi-square test was used to compare categorical variables. The Wilcoxon rank-sum test was used to compare continuous variables between the two groups. The Wilcoxon signed-rank test was used to compare continuous variables between paired groups. Variable candidates for multiple logistic regression analyses were those at admission with significant differences in the Wilcoxon rank-sum test between Day-7 FBG < 7.0 and Day-7 FBG ≥ 7.0 groups. We defined multicollinearity as VIF of 3 or more. For assessment of multicollinearity, a dummy variable was used to represent categorical data, such as data on the use of medication or not. We used variables without multicollinearity and conducted multiple logistic regression analyses to identify independent variables that distinguish between the Day-7 FBG < 7.0 and Day-7 FBG ≥ 7.0 groups. We estimated the cut-off values of independent variables for the Day-7 FBG < 7.0 group using the area under the curve values derived from receiver operating characteristic curves.

Statistical significance was set at *p* <0.05. We used JMP software (version 16.1; SAS Institute, Cary, NC, USA) for all statistical analyses. 

## 5. Presentation

We presented a part of this paper at the AHA International Stroke Conference 2018 (ATP368).

## Figures and Tables

**Table 1:** Patient characteristics (N = 51).

Variable	Value
Ischemic, n (%)	48 (94.1%)
Sex, n (%)	Male 37 (72.5%), Female 14 (27.5)
Age, y	71 (63, 77)
BMI, kg/m^2^	24.3 (22.2, 27.2)
BH, m	1.64 (1.60, 1.69)
BW, kg	65 (58, 78)
Alb, g/L	41 (39, 44)
Cre, mmol/L	72.5 (57.5, 86.6)
eGFR, mL·min^−1^·m^−2^ BSA	68.2 (56.5, 83.5)
HbA1c, % (NGSP)	9.3 (7.6, 11.0)
Hct, %	43.1 (40.2, 44.7)
Glucose, mmol/L	14.54 (12.82, 17.43)
TC, mmol/L	5.35 (4.73, 6.36)
LDL, mmol/L	3.28 (2.67, 3.94)
HDL, mmol/L	1.33 (1.07, 1.74)
TG, mmol/L	1.51 (0.89, 2.17)
hs-CRP, µg/L	1900 (600, 4000)
NIHSS at admission	2 (1, 4)
NIHSS at discharge	1 (0, 3)
Hospitalization, days	8 (8, 9)
Discharge to home, n (%)	23 (45.1%)

All values except for categorical data are presented as median (interquartile range). Alb, albumin; BH, body height; BMI, body mass index; BSA, body surface area; BW, body weight; Cre, creatinine; eGFR, estimated glomerular filtration rate; HbA1c, glycated hemoglobin; Hct, hematocrit; HDL, high-density lipoprotein cholesterol; hs-CRP, high-sensitivity C-reactive protein; LDL, low-density lipoprotein cholesterol; NGSP, National Glycohemoglobin Standardization Program; n, number; NIHSS, National Institutes of Health Stroke Scale score; TC, total cholesterol; TG, triglyceride.

**Table 2:** Diet, carbs, glucose level, and urine test.

N	51
Energy-restricted diet, kcal	1400 (1200, 1400)
Carb restriction, g	140 (120, 160)
BG adm, mmol/L	14.5 (12.8, 17.4)
Day-3 FBG, mmol/L	8.9 (7.4, 10.3)
Day-7 FBG, mmol/L	6.6 (5.8, 7.8)
Day-7 FBG < 7.0 mmol/L, n (%)	33 (64.7%)
Hct adm, %	43.1 (40.2, 44.7)
Day-7 Hct, %	40.6 (39.1, 42.9)
U-glu score adm	3.5 (1.6, 4)
U-glu score on day 7	4 (4, 4)
U-ketone score adm	0 (0, 0)
U-ketone score on day 7	2 (0, 3)

All values except for categorical data are presented as median (interquartile range). adm, at admission; Carb, carbohydrate; BG, blood glucose; Day-3 FBG, FBG level on day 3; Day-7 FBG, FBG level on day 7; Day-7 Hct, hematocrit on day 7; FBG, fasting blood glucose; Hct, hematocrit; N, number; U-glu, urine glucose; U-ketone, urine ketones.

**Table 3:** Changes in blood glucose level (N = 51).

N	At Admission		On Day 3		On Day 7
BG, mmol/L	14.5 (12.8, 17.4)	→	8.9 (7.4, 10.3)	→	6.6 (5.8, 7.8)
*p*-value		**<0.0001**		**<0.0001**	

Boldface indicates significance. All values are presented as median (interquartile range). BG, blood glucose.

**Table 4:** Changes in Hct level, glucose level, and urine test (N = 51).

N	At Admission	On Day 7	*p*-Value
BG adm, mmol/L	14.5 (12.8, 17.4)	6.6 (5.8, 7.8)	**<0.0001**
Hct, %	43.1 (40.2, 44.7)	40.6 (39.1, 42.9)	**0.0072**
U-glu score	3.5 (1.6, 4)	4 (4, 4)	**<0.0001**
U-ketone score	0 (0, 0)	2 (0, 3)	**<0.0001**

Boldface indicates significance. All values are presented as median (interquartile range). BG, blood glucose; Hct, hematocrit; U-glu, urine glucose; U-ketone, urine ketones.

## Data Availability

The datasets generated and/or analyzed during the current study are available from the corresponding author upon reasonable request bacause of its usage in the ongoing study.

## Supplementary Materials

The following are available online at https://www.mdpi.com/article/10.3390/metabo12010025/s1, Figure S1: Serial changes in blood glucose levels in the Day-7 FBG < 7.0 and Day-7 FBG ≥ 7.0 groups, Table S1: Stroke subtypes, Table S2: Diabetes medications used prior to admission, Table S3: Total energy-restriction diet for hospitalization, Table S4: SGLT2i use during hospitalization, Table S5: Diabetes medications except for SGLT2i used during hospitalization, Table S6: Evaluation of groupwise changes in blood glucose levels by the Wilcoxon rank-sum test, Table S7: Evaluation of groupwise changes in blood glucose levels by the Wilcoxon signed-rank test, Table S8: Intergroup comparison of age, male sex, BMI, and biomarkers at admission, Table S9: Intergroup comparison of DPP4, SU, Big, *α*GI, and SGLT2i use prior to admission, Table S10: Intergroup comparison of diabetes medications used during hospitalization except for SGLT2i, Table S11: Intergroup comparison of DPP4, Big, *α*GI, and LIA used during hospitalization, Table S12: Variance inflation factor of variables for multiple logistic regression analysis, Table S13: Multiple logistic regression for Day-7 FBG.
