# Supplementary material for: Safety and Effectiveness of Sodium–Glucose Cotransporter 2 Inhibitor Combined with Medical Nutrition Therapy for Hyperglycemia in Acute Stroke: A Retrospective Study
Source: Metabolites. 2021 Dec 28;12(1):25. doi: 10.3390/metabo12010025 (PMC8779243; doi:10.3390/metabo12010025)
Supplement: Supplementary file 1 [file metabolites-12-00025-s001.zip › metabolites-1524307-supplementary.pdf]

Supplementary Materials

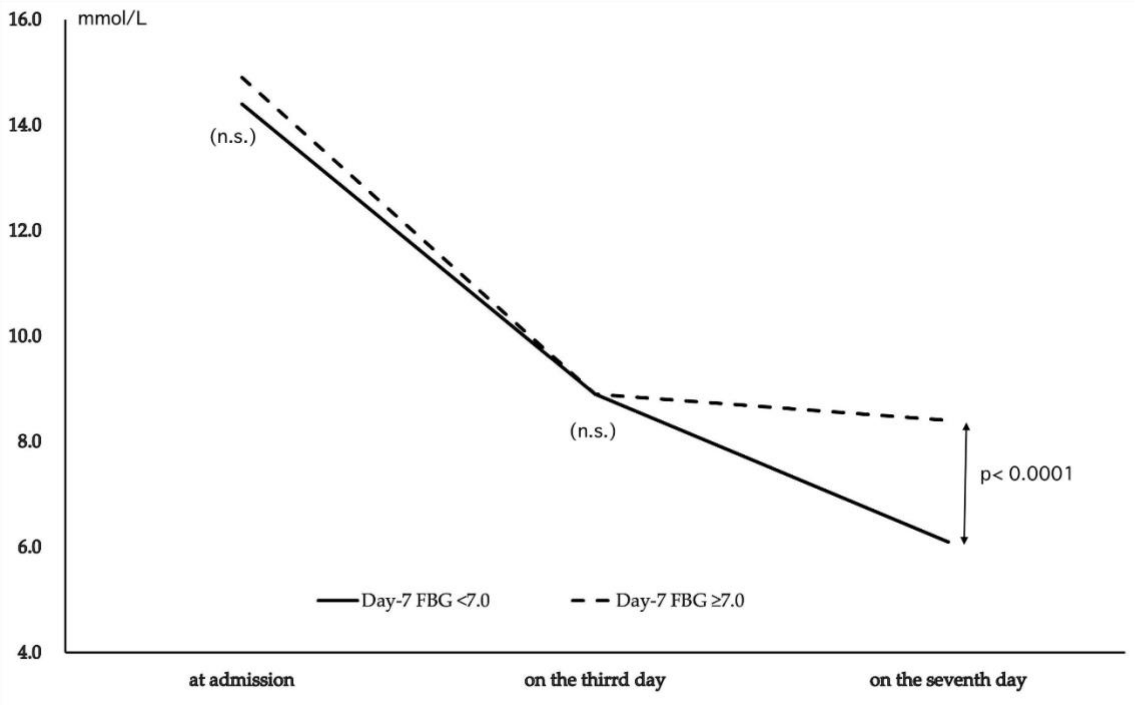

Figure S1. Serial changes of blood glucose levels in the Day-7 FBG <7.0 and Day-7 FBG ≥7.0 groups. FBG, fasting blood glucose; n.s., not significant; p, probability

Table S1. Stroke subtypes.

Classification	N
Transient ischemic attack	1
Small-vessel occlusion (lacune)	19
Large-artery atherosclerosis	17
Cardiogenic stroke	3
Stroke of undetermined etiology	8
Hypertensive intracerebral hemorrhage	3

N, number

Table S2. Diabetes medications used prior to admission.

N	51 (100%)
None	28 (54.9%)
DPP4	2 (3.9%)
SU	4 (7.8%)
Big	1 (2.0%)
Big+SU	3 (5.9%)
DPP4+Big	6 (11.8%)
DPP4+SU	2 (3.9%)
DPP4+Big+SU	3 (5.9%)
DPP4+SU+SGLT2i	1 (2.0%)
DPP4+Big+SU+αGI	1 (2.0%)

αGI, alpha-glucosidase inhibitor; Big, biguanide; DPP4, dipeptidyl peptidase-4 inhibitors; N, number; SGLT2i, sodium-glucose cotransporter 2 inhibitors; SU, sulfonylurea

Table S3. Total energy-restriction diet for hospitalization.

Target total energy	A total energy-restricted diet
1,000 kcal ≤ target total energy < 1,200 kcal	1,000 kcal/day
1,200 kcal ≤ target total energy < 1,400 kcal	1,200 kcal/day
1,400 kcal ≤ target total energy < 1,600 kcal	1,400 kcal/day
1,600 kcal ≤ target total energy < 1,800 kcal	1,600 kcal/day
1,800 kcal ≤ target total energy < 2,000 kcal	1,800 kcal/day

Table S4. SGLT2i use during hospitalization.

SGLT2i	N
Luseogliflozin hydrate 2.5 mg per day	26
Dapagliflozin propylene glycolate hydrate 5 mg per day	17
Canagliflozin hydrate 100 mg per day	8
Total	51

N, number; SGLT2i, sodium-glucose cotransporter 2 inhibitors

Table S5. Diabetes medications except for SGLT2i used during hospitalization.

N	51 (100%)
None	9 (17.7%)
DPP4	16 (31.4%)
Big	1 (2.0%)
LIA	1 (2.0%)
DPP4+Big	11 (21.6%)
DPP4+ αGI	3 (5.9%)
DPP4+LIA	4 (7.8%)
DPP4+Big+αGI	3 (5.9%)
DPP4+Big+LIA	3 (5.9%)

αGI, alpha-glucosidase inhibitor; Big, biguanide; DPP4, dipeptidyl peptidase-4 inhibitors; LIA, long-acting insulin analog; N, number; SGLT2i, sodium-glucose cotransporter 2 inhibitors

Table S6. Evaluation of groupwise changes in blood glucose levels by the Wilcoxon rank-sum test.

	Day-7 FBG <7.0	Day-7 FBG ≥7.0	*P*-value
BG at admission, mmol/L	14.4 (12.4, 17.1)	14.9 (12.5, 17.1)	n.s.
FBG on day 3, mmol/L	8.9 (7.3, 10.6)	8.9 (7.3, 10.4)	n.s.
FBG on day 7, mmol/L	6.1 (5.1, 6.5)	8.4 (7.5, 9.1)	**< 0.0001**

All values are presented as median (interquartile range). Boldface indicates significance. BG, blood glucose; Day-7 FBG, FBG level on day 7; FBG, fasting blood glucose; n.s., not significance; *P*, probability

Table S7. Evaluation of groupwise changes in blood glucose levels by the Wilcoxon signed-rank test.

Group	At admission		On day 3		On day 7
Day-7 FBG <7.0, mmol/L	14.4 (12.4, 17.1)	→	8.9 (7.3, 10.6)	→	6.1 (5.1, 6.5)
P-value		**< 0.0001**		**< 0.0001**	
Day-7 FBG ≥7.0, mmol/L	14.9 (12.5, 17.1)	→	8.9 (7.3, 10.4)	→	8.4 (7.5, 9.1)
P-value		**< 0.0001**		ns	

All values are presented as median (interquartile range). Boldface indicates significance.

FBG, fasting blood glucose

Table S8. Intergroup comparison of age, male sex, BMI, and biomarkers at admission.

	Day-7 FBG <7.0	Day-7 FBG ≥7.0	*P*-value
N	33	18	
Age, years	69 (62.5, 76)	74 (66.5, 79.3)	**0.0473**
Sex, n (%)	Male:25 (75.8%)	Male:12 (66.7%)	0.4905
	Female:8 (24.2%)	Female:6 (33.33%)	
BMI, kg/m^2	24.5 (23, 27.4)	23.4 (20.5, 27.3)	0.3696
Cre, mmol/L	72.5 (56.6, 83.5)	70.3 (60.1, 91.3)	0.5345
eGFR, $mL \cdot min^{-1} \cdot m^{-2}$ BSA	68.4 (63.0, 85.2)	65.2 (47.3, 81.8)	0.2489
hs-CRP, μg/L	1900 (500, 4750)	1800 (775, 3150)	0.9528

Boldface indicates significance. BMI, body mass index; BSA, body surface area; Cre, creatinine; Day-7 FBG, FBG level on day 7, mmol/L; eGFR, estimated glomerular filtration rate; FBG, fasting blood glucose; hs-CRP, high-sensitivity C-reactive protein; N, number, *P*, probability

Table S9. Intergroup comparison of DPP4, SU, Big, αGI, and SGLT2i use prior to admission.

	Day-7 FBG <7.0, mmol/L	Day-7 FBG ≥7.0, mmol/L	*P*-value
N	33	18	
DPP4	7	8	0.0856
No DPP4	26	10	
SU	5	9	**0.0085**
No SU	28	9	
Big	8	6	0.4905
No Big	25	12	
αGI	1	0	0.3478
No αGI	32	18	
SGLT2i	0	1	0.1454
No SGLT2i	33	17	

Boldface indicates significance.

αGI, alpha-glucosidase inhibitor; Big, biguanide; Day-7 FBG, FBG level on day 7; DPP4, dipeptidyl peptidase-4 inhibitors; FBG, fasting blood glucose; N, number; SU, sulfonylurea

Table S10. Intergroup comparison of diabetes medications used during hospitalization except for SGLT2i.

	Day-7 FBG <7.0, mmol/L	Day-7 FBG ≥7.0, mmol/L	N (%)
N	33	18	51 (100%)
None	8	1	9 (17.6%)
DPP4	11	5	16 (31.4%)
Big	1	0	1 (2.0%)
DPP4+Big	7	4	11 (21.6%)
DPP4+αGI	1	2	3 (5.9%)
DPP4+Big+αGI	1	2	3 (5.9%)
LIA	1	0	1 (2.0%)
DPP4+LIA	2	2	4 (7.8%)
DPP4+Big+LIA	1	2	3 (5.9%)

αGI, alpha-glucosidase inhibitor; Big, biguanide; Day-7 FBG, FBG level on day 7; DPP4, dipeptidyl peptidase-4 inhibitors; FBG, fasting blood glucose; LIA, long-acting insulin analog; N, number

Table S11. Intergroup comparison of DPP4, Big, αGI, and LIA used during hospitalization.

	Day-7 FBG <7.0, mmol/L	Day-7 FBG ≥7.0, mmol/L	*P*-value
N	33	18	
DPP4	23	17	**0.0257**
No DPP4	10	1	
Big	10	8	0.3155
No Big	23	10	
αGI	2	4	0.0951
No αGI	31	14	
LIA	4	4	0.3519
No LIA	29	14	

Boldface indicates significance.

αGI, alpha-glucosidase inhibitor; Big, biguanide; Day-7 FBG, FBG level on day 7; DPP4, dipeptidyl peptidase-4 inhibitors; FBG, fasting blood glucose; LIA, long-acting insulin analog; N, number

Table S12. Variance inflation factor of variables for multiple logistic regression analysis.

	VIF
Age	1.07
SU use	1.07

SU, sulfonylurea; VIF, variance inflation factor

Table S13. Multiple logistic regression for Day-7 FBG<7.0 using receiver operating characteristic curves (N=676).

	Odds ratio	*P*-value	AUC	BIC
		0.0084	0.736	68.5
Age	0.94 (0.86, 1.01)	0.1044		
SU use (0=no SU use, 1= SU use)	0.23 (0.05, 0.86)	**0.0290**		

AUC, area under the curve; BIC, Bayesian information criterion; Day-7 FBG, FBG level on day 7; FBG, fasting blood glucose; *P*, probability; SU, sulfonylurea
